# Glove Port Technique for Transanal Endoscopic Microsurgery

**DOI:** 10.1155/2012/383025

**Published:** 2012-06-03

**Authors:** Carrara Alessandro, Mangiola Daniela, Motter Michele, Tirone Andrea, Ghezzi Gianmarco, Silvestri Massimo, Zappalà Orazio, Gasperetti Fabio, Tirone Giuseppe

**Affiliations:** ^1^1st Division of General Surgery, S. Chiara Hospital, Largo Medaglie d'Oro 1, 38100 Trento, Italy; ^2^Division of Medical Oncology and Palliative Medicine, Policlinic G.B. Rossi, 37134 Verona, Italy; ^3^2nd Division of General Surgery, Policlinic “Le Scotte”, 53100 Siena, Italy

## Abstract

*Introduction*. Despite initial enthusiasm, the use of transanal endoscopic microsurgery (TEM) is still quite limited at present because of the expense of highly specialized equipment and the complexity of the learning curve. Furthermore, some authors report a relevant, although temporary, effect on anorectal function because of the considerable anal dilatation which can even produce a rupture of the internal anal sphincter. The “glove TEM” proposes itself as an alternative to traditional TEM that could settle these problems. *Materials and Methods*. The technique is accurately described together with the necessary equipment to perform it. Between 2011 and 2012, we operated eight patients with this technique for rectal adenomas or early carcinomas achieving R0 resection in all cases and reporting no early or late complications during the first five months of followup. *Discussion*. This technique offers multiple advantages compared to the original TEM. (i) It allows the use of all available laparoscopic instruments. (ii) It gives a great manoeuvrability of the instruments in contrast to rigid rectoscope systems. (iii) Given the limited length of the device, it permits to operate on tumors closer to the dentate line. (iv) It is less traumatic to the anal sphincter. It is definitively much cheaper. *Conclusions*. We believe that this new technique is easy to perform, cost-effective, and less traumatic to the anal sphincter compared to traditional TEM.

## 1. Introduction

The transanal endoscopic microsurgery (TEM), originally designed by Buess et al., is a safe and minimally invasive surgical technique for the treatment of benign adenomas and early-stage carcinomas of the low, middle, and upper rectum not amenable to traditional colonoscopic excision [1, 2]. TEM satisfies two major aims: complete removal of the lesion and maintenance of sphincter function. Additionally, TEM offers the benefit of avoiding the trauma and morbidity of the conventional open surgery major leading to a better quality of life for the patient, less postoperative hospital stay, and reduced morbidity and mortality rates. The TEM procedure involves a transanal approach using a set of endoscopic surgical instruments that can reach further into the rectum than other forms of local excision together with a form of enhanced vision. The excellence of the image allows for more precise excision; according to many authors, this implies a better oncologic outcome and a lower reoperation rate [3, 4]. Following our experience with the laparoscopic single-port surgery (SILS) and particularly with a homemade device composed of a disposable wound retractor (Alexis) and a simple surgical glove [5], we recently started to use the same device for transanal endoscopic surgery. In this paper, we describe this technique, reporting the results of our first eight cases.

## 2. Materials and Methods

**Figure 1:**
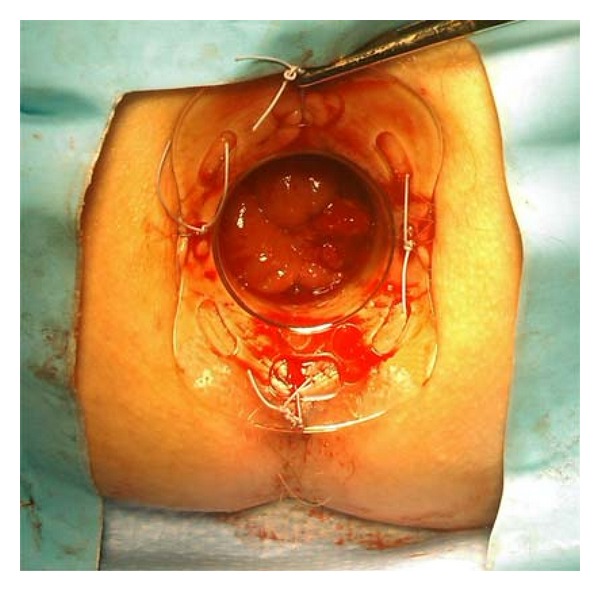
The disposable anal retractor is well fixed with four skin stitches.

**Figure 2:**
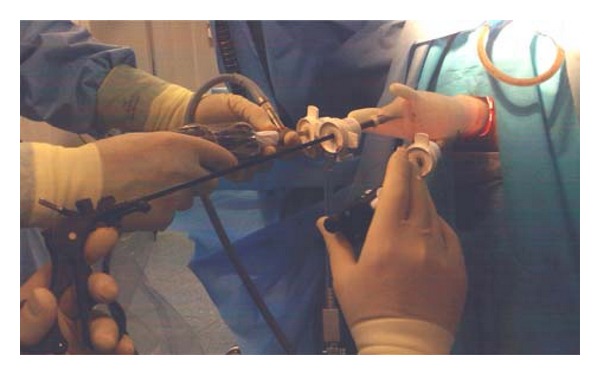
The laparoscopic camera is inserted via the middle finger port.

**Figure 3:**
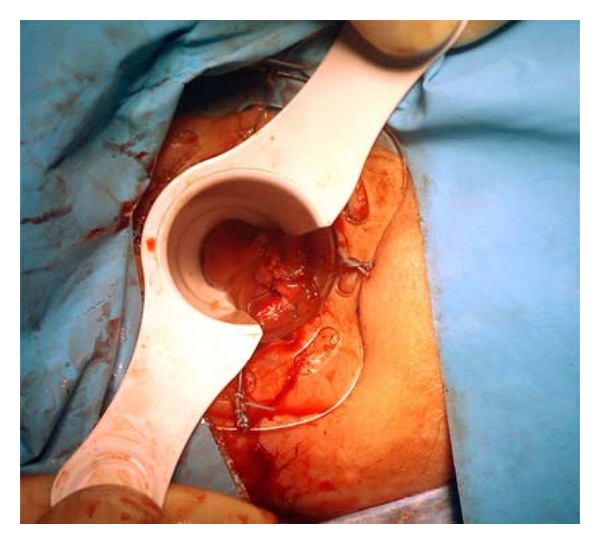
Final result after suturing the excisional area.

We use a wound retractor (Alexis) applied through a disposable circular anal retractor (Sapimed SpA) well fixed with skin stitches (Figure 1). A powder-free surgical glove is then put, air tight, on the wound retractor, and three or four trocars are inserted via the finger tips. A laparoscopic camera is inserted via the middle finger port (Figure 2). All laparoscopic standard instruments can be used without any bond or limitation in maneuverability since they are free to work through the wound retractor. The pneumorectum is maintained at almost 12 mmHg. The operation then proceeds exactly like in the traditional TEM, with the mucosal marking all around the lesion. The tumor is then resected dissecting the rectal wall along the marking deeply to the mesorectal tissue preserving wide safety margins all around the lesion. The smaller length of the anal retractor, compared to the traditional TEM, allows easily excising the distal margin of the specimen even at only 1.5/2 cm from the dentate line. The excisional area is then closed with an absorbable continue suture (Figure 3).

## 3. Results

We recently used this technique on eight patients, five large rectal adenomas, two T2 cancers of the proximal rectum not amenable for abdominal surgery, and one T1 cancer of the distal rectum, achieving R0 resection in all cases. The average distal margin from the anal verge was 6,5 cm (range 1,5–12 cm). The hospital stay was short, with all patients discharged in the first postoperative day. No early or late complications were reported during the first five months of followup.

## 4. Discussion

The TEM approach offers patients with rectal lesions, an additional treatment option with several advantages: the major benefits of TEM include avoidance of a major abdominal operation, avoidance of a colostomy, visualization improved over that of customary transanal approach, and ability to expand transanal excision proximally. Nevertheless, despite these advantages, the use of TEM is still quite limited. The reasons for this unrealized potential are to be found mostly in the high cost of the equipment and in the complexity of the learning curve. The risks of TEM and local excision may include infection, bleeding, and perforation into the peritoneal cavity or vagina. These are fortunately rare but would require further surgery. Furthermore, some authors report a relevant, although temporary, effect on anorectal function because of the considerable anal dilatation due to the rectoscope wide diameter (40 mm wide). Gracia Solanas et al. found TEM procedure can result in a rupture of the internal anal sphincter (25% of cases on a casistic of 40 patients that), with the consequent decreasing in anal resting pressure, and in a dilatation without rupture of external sphincter what produces a decreasing of maximal squeeze pressure. The fall of anal pressures had minimal clinical repercussion when the sphincter is intact, but, when the internal anal sphincter is broken, a temporal incontinence develops [6]. In another study conducted by Herman et al. [7], the effects of TEM on anorectal motility and function are investigated. The authors report the results of anorectal motility studies (using pull-through anorectal manometry and rectal barostat) and endoanal ultrasound prior to surgery and 3 weeks and 6 months after TEM on 33 patients with rectal tumors. The authors conclude that TEM has relevant but temporary effects on anorectal motility; nevertheless, only few motility disturbances are reflected in continence results such as liquid stool/flatus incontinence, soiling, stool frequency, and urgency (21%), since 79% of patients following TEM reported perfect continence control.

**Figure 4:**
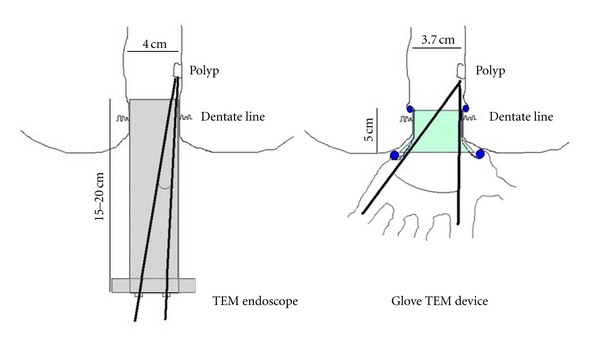
Traditional TEM endoscope versus Glove TEM device: dimensions and instruments working angles.

In our opinion, the glove TEM technique offers multiple advantages:

1. the possibility of using almost all available laparoscopic instruments with the maximum range of maneuverability in contrast to rigid and long rectoscope systems. As is shown in Figure 4 traditional TEM endoscope is 15–20 centimeters long depending on the model; the anal dilator utilized in the Glove TEM is only 5 centimeters long allowing to operate with a broader angle between the instruments especially when the lesion is in the lower rectum;
2. the ability to operate on tumors closer to the dentate line till a minimum distance of 1,5/2 cm given the above-mentioned limited length of the Glove TEM device together with its great handiness;
3. minor trauma to the anal sphincter due to the smaller size of the retractor which is only 3.7 centimeters compared to the 4 centimeters of the TEM endoscope. Although the two devices have a variation of no more than 3 mm in diameter, this entails a difference of almost 1 centimeter in their circumferences (11,61 cm versus 12,56 cm) which we believe could be sufficient to consider the glove TEM system less stressful to the anorectal function. Furthermore, even though our casistic is too small to gain statistical significance, we have to note that we did not report any clinically detectable anorectal dysfunction on the patients we operated with the Glove TEM;
4. last but not least, the glove TEM is much cheaper than the traditional TEM [8] because it can be performed with the usual laparoscopic multiuse equipment through a simple homemade device whose cost is approximately 100 USD (one disposable anal dilator, one Alexis wound retractor, and a surgical glove).

Nevertheless, we have to report that some pitfalls emerged in our initially experience with this technique. Firstly, we strongly suggest to use thin (5 mm) and long laparoscopic camera (50 centimetres) in addiction to three slim trocars inserted through finger tips to avoid conflicts between instruments during the operation. Secondly, a hand support of the trocars and a visual assistance are necessary during each introduction and extraction of the laparoscopic instruments since the glove's flexibility and elasticity make these operations extremely troublesome and expose the glove to the risk of accidental perforation with consequent gas leakage. New devices developed for laparoscopic SILS (GelPOINT, OCTO Port) in substitution of the glove technique could probably settle these problems.

## 5. Conclusions

On the basis of our early experience, we believe that glove TEM is a promising surgical technique, safe, effective, and easy to install and to perform. It is made from commonly used and relatively inexpensive surgical equipment and offers the possibility to use all the conventional laparoscopic instruments with an amazing manoeuvrability thus avoiding long and complex learning curves for a laparoscopic surgeon. Our experience demonstrates that this technique can allow use of transanal endoscopic microsurgery in a broader spectrum of patients than maybe otherwise possible for economic and technical reasons.

## References

[1] Buess G, Mentges B, Manncke K, Starlinger M, Becker HD (1991). Minimal invasive surgery in the local treatment of rectal cancer. *International Journal of Colorectal Disease*.

[2] Léonard D, Colin J-F, Remue C, Jamart J, Kartheuser A (2012). Transanal endoscopic microsurgery: long-term experience, indication expansion, and technical improvements. *Surgical Endoscopy and other Interventional Techniques*.

[3] Middleton PF, Sutherland LM, Maddern GJ (2005). Transanal endoscopic microsurgery: a systematic review. *Diseases of the Colon and Rectum*.

[4] Dias AR, Nahas CSR, Marques CFS, Nahas SC, Cecconello I (2009). Transanal endoscopic microsurgery: indications, results and controversies. *Techniques in Coloproctology*.

[5] Ishida H, Okada N, Ishibashi K, Ohsawa T, Kumamoto K, Haga N (2011). Single-incision laparoscopic-assisted surgery for colon cancer via a periumbilical approach using a surgical glove: initial experience with 9 cases. *International Journal of Surgery*.

[6] Gracia Solanas JA, Ramírez Rodríguez JM, Aguilella Diago V, Elía Guedea M, Martínez Díez M (2006). A prospective study about functional and anatomic consequences of transanal endoscopic microsurgery. *Revista Espanola de Enfermedades Digestivas*.

[7] Herman RM, Richter P, Walȩga P, Popiela T (2001). Anorectal sphincter function and rectal barostat study in patients following transanal endoscopic microsurgery. *International Journal of Colorectal Disease*.

[8] Van Den Boezem PB, Kruyt PM, Stommel MWJ, Tobon Morales R, Cuesta MA, Sietses C (2012). Transanal single-port surgery for the resection of large polyps. *Digestive Surgery*.

